# Increased Oxidative and Nitrative Stress Accelerates Aging of the Retinal Vasculature in the Diabetic Retina

**DOI:** 10.1371/journal.pone.0139664

**Published:** 2015-10-14

**Authors:** Folami Lamoke, Sean Shaw, Jianghe Yuan, Sudha Ananth, Michael Duncan, Pamela Martin, Manuela Bartoli

**Affiliations:** 1 Dept. of Ophthalmology, Medical College of Georgia, Georgia Regents University, Augusta, Georgia, United States of America; 2 Department of Biochemistry and Molecular Biology, Medical College of Georgia, Georgia Regents University, Augusta, Georgia, United States of America; 3 Dept. of Medicine, Section of Gastroenterology/Hepatology, Medical College of Georgia, Georgia Regents University, Augusta, Georgia, United States of America; Cedars-Sinai Medical Center; UCLA School of Medicine, UNITED STATES

## Abstract

Hyperglycemia-induced retinal oxidative and nitrative stress can accelerate vascular cell aging, which may lead to vascular dysfunction as seen in diabetes. There is no information on whether this may contribute to the progression of diabetic retinopathy (DR). In this study, we have assessed the occurrence of senescence-associated markers in retinas of streptozotocin-induced diabetic rats at 8 and 12 weeks of hyperglycemia as compared to normoglycemic aging (12 and 14 months) and adult (4.5 months) rat retinas. We have found that in the diabetic retinas there was an up-regulation of senescence-associated markers SA-**β-**Gal, p16^INK4a^ and miR34a, which correlated with decreased expression of SIRT1, a target of miR34a. Expression of senescence-associated factors primarily found in retinal microvasculature of diabetic rats exceeded levels measured in adult and aging rat retinas. In aging rats, retinal expression of senescence associated-factors was mainly localized at the level of the retinal pigmented epithelium and only minimally in the retinal microvasculature. The expression of oxidative/nitrative stress markers such as 4-hydroxynonenal and nitrotyrosine was more pronounced in the retinal vasculature of diabetic rats as compared to normoglycemic aging and adult rat retinas. Treatments of STZ-rats with the anti-nitrating drug FeTPPS (10mg/Kg/day) significantly reduced the appearance of senescence markers in the retinal microvasculature. Our results demonstrate that hyperglycemia accelerates retinal microvascular cell aging whereas physiological aging affects primarily cells of the retinal pigmented epithelium. In conclusion, hyperglycemia-induced retinal vessel dysfunction and DR progression involve vascular cell senescence due to increased oxidative/nitrative stress.

## Introduction

Hyperglycemia-induced dysfunction of retinal blood vessels is a major contributing factor in the pathogenesis of diabetic retinopathy (DR), the leading cause of blindness in working-age adults [1–3]. Despite the recent evidence suggesting the existence of both neural and vascular alterations in the diabetic retina [4–7], hyperglycemia-induced retinal microangiopathy remains a main pathogenic event for DR and a key therapeutic target for its prevention and cure [8, 9].

Several molecular mechanisms have been implicated to explain hyperglycemia-induced retinal vascular dysfunction. In particular, augmented oxidative and nitrative stress, due to increased production of reactive oxygen and nitrogen species (ROS and RNS, respectively) [10, 11] and impaired endogenous antioxidant ability [12], have been shown to induce inflammatory responses leading to capillary cell dysfunction and death [10].

Oxidative stress-induced vascular inflammation also occurs during physiological aging [13–16] where vascular senescence plays a key role in the pathogenesis of age-associated cardiovascular disease [17–21]. Interestingly, increased oxidative and nitrative stress may accelerate vascular senescence also in diabetes [22–24]. As a result, endothelial cells (ECs) and surrounding tissues undergo structural alterations in a complex senescence process characteristically similar to what occurs during physiological aging [25–29], but not including replicative senescence-associated telomere shortening and its downstream consequences [30].

The acquisition of senescence-like features in blood vessels can promote a chronic inflammatory phenotype known as senescence-associated secretory phenotype (SASP) [31], characterized by up-regulation of inflammatory cytokines largely due to persistent acetylation/activation of the pro-inflammatory transcription factor NF-kB [32].

Here we have investigated the effects of hyperglycemia in promoting/accelerating aging of the retinal microvasculature by monitoring the appearance of senescence-like markers relative to oxidative/nitrative stress parameters in diabetic adult rats (4.5 months old) at 8 and 12 weeks of hyperglycemia and in aging non-diabetic rats (12–14 months).

The obtained results show that hyperglycemia-induced retinal microangjopathy involves accelerated senescence of the retinal microvasculature resulting from increased oxidative and nitrative stress and from induction of redox-dependent intracellular signaling and epigenetic events.

## Materials and Methods

### Animals

All animals were housed in the vivarium of Georgia Regents University and kept under a 12 hour day/night light cycle. Adult male Sprague-Dawley (SD) rats (250–300g) obtained from Harlan Laboratories (Dublin, VA) were made diabetic by a single intravenous injection of streptozotocin (STZ) [65mg/kg dissolved in 0.1M sodium citrate (pH 4.5)]. Control rats from the same strain (SD) were delivered equal volumes of the vehicle alone. Rats were considered to be diabetic when fasting blood glucose levels were found to be ≥300 mg/dL. One group of STZ-rats kept diabetic for 8 weeks were treated with daily doses (10mg/Kg/day) of the peroxynitrite decomposition catalysts 5,10,15,20-tetrakis(4-sulfonatophenyl) porphyrinato iron III chloride (FeTPPS), administered in the drinking water [33]. FeTPPS prevents the formation of nitrotyrosine by scavenging out peroxynitrite and also limits the levels of hydroxyl radicals produced as peroxynitrite by-products [34, 35]. All the diabetic rats were sacrificed after 8 and 12 weeks of hyperglycemia with an overdose of anesthesia followed by a thoracotomy.

Another set of animals used in our experiments included non-diabetic rats at 12 and 14 months of age, which represented the aging group. Normoglycemic rats at 4.5 months of age (age-matched with the STZ-rats) were used as controls.

At the time of the sacrifice, retinas were excised and preserved in different conditions according to the subsequent biochemical and morphological analysis. A list of the different experimental groups is provided in Table 1.

**Table 1:** Experimental animal groups and metabolic parameters.

Treatment Group	Duration of Diabetes	Age in months (mos)	n	Weight (g)	Blood Glucose (mg/dl)
**Control**	0	4.5	6	315.6	187.6±12.9
**D**_**8wks**_	8 wks	4	6	278.2*	456.8±30.8
**D**_**12wks**_	12 wks	5	6	267.1*	464.9±26.2
**D**_**8wks**_ **+FeTPPS**	8 wks	4	6	282.6	453.3±22.1
**A**_**12mos**_	0	12	6	374.2°	176±10.9
**A**_**14mos**_	0	14	6	387.5°	182±11.3

### Morphological analysis

Eyes were enucleated, embedded in Optimal Cutting Temperature (OCT) mounting medium (Tissue Tek, Torrance, CA), and frozen on dry ice. Retinal cryosections (20 **μ**M) were fixed in 4% paraformaldehyde (PFA) prior to immunohistochemical analysis. For retinal and RPE flat mounts, enucleated globes were immediately fixed in 4% PFA followed by retinal extraction and separation from RPE. Fixed retinal sections and whole retinas were incubated overnight at 4°C with primary antibodies, rabbit anti-CDKN2A/p16^INK4a^ (1:500, Abcam, Cambridge, MA), rabbit anti-SIRT1 (1:100, Cell Signaling, Danvers, MA), mouse anti-nitrotyrosine (Cayman Chemical, Ann Arbor, MI), or goat anti-4-hydroxynonenal (1:200, Abcam, Cambridge, MA) and co-labeled with isolectin B4 (1:1000, Invitrogen, Grand Island, NY) to localize retinal vascular structures. Slides/retinal flat mounts were washed with 1% Triton X-100 in 0.1M PBS (pH 7.4) 3 times followed by a one hour incubation with the secondary antibodies, goat anti-rabbit IgG-conjugated Alexa Fluor 488 and goat anti-mouse IgG-conjugated Alexa Fluor 488, chicken anti-goat Alexa Fluor 488, and streptavidin. Nuclei were stained following 5 minute incubation with Hoescht 33342 (Invitrogen, Carlsbad, CA) in phosphate buffered saline (PBS) at a1:24,000 dilution. Slides and retinal flat mounts were then mounted using Fluoromount (Fisher Scientific, Pittsburg, PA) or Vectashield (Vector Laboratories, Burlingame, CA). For flat mounts, retinas were sectioned into four quadrants and flattened upon Superfrost microscope slides (Fisher Scientific, Pittsburgh, PA, USA). Sections and flat mounts were examined by epifluorescence using a Zeiss Axioplan-2 microscope (Carl Zeiss, Göttingen, Germany) equipped with the Axiovision program (version 4.7).

### Senescence-associated β-galactosidase activity assay

Senescence-associated **β**-galactosidase (SA-**β**-Gal) reactivity-based assay was performed to evidence senescent areas in retinal sections, retinal flat mounts, and RPE flat mounts using a commercially available kit assay (Cell Signalling, Danver, MA). The tissues were fixed with 2% formaldehyde and 0.2% glutaraldehyde in PBS. Positive reactivity to **β**- galactosidase is evidenced at pH 6 only in senescent cells, *in vitro* and *in vivo*. Slides and/or flat mounts (retinas and RPE) images were captured 20X and 63X magnification by light microscopy using Zeiss Axioplan2 (Carl Zeiss Microscopy, Thornwood, NY).

### Protein analysis

Western blotting analysis was performed according to standard protocols [36] using anti-SIRT1 (Cell Signaling, Danvers, MA) and anti-p16^INK4a^ (Abcam, Cambridge, MA). After incubation with horseradish peroxidase-conjugated secondary antibody (GE Healthcare Life Sciences, Pittsburg, PA) bands were detected using the enzymatic chemiluminescence reagent ECL (GE Healthcare, Pittsburg, PA).

### Sirt-1 activity assay

An assay for the detection of SIRT1 decatylase activity in retinal tissue was performed as a two-step enzymatic reaction as per manufacturer’s instructions (Sigma-Aldrich, St. Louis, MO). In the first step, deacetylation by SIRT1 is performed using a substrate that contains an acetylated lysine side chain. In the next step, cleavage of the deacetylated substrate by the Developing Solution occurred resulting in the release of a highly fluorescent group. The measured fluorescence at 340nm/430nm wavelength (excitation/emission) using spectraMax Gemini EM, (Molecular Devices, Sunnydale, CA) was directly proportional to the deacetylation activity of the enzyme in the sample.

### mRNA analysis

QiagenRNeasy extraction kit was used to extract mRNA from rat retinas. Quantification of SIRT1 and p16^INK4a^ mRNA expression was performed using Quantitative Real-time RT-PCR. All primers listed in Table 2 were obtained from Invitrogen (Carlsbad, CA). All data were normalized to **β** -actin mRNA.

**Table 2:** List of Primers.

gene	Primer sequence
**p16** ^**INK4a**^	Sense:TGGACAATGGCTACTCAA
	Antisense: TTCCCTGAG ACACTAGAT
**SIRT1**	Sense:TGTTTCCTGTGGGATACCTGA
	Antisense:TGAAGAATGGTCTTGGGTCTTT
**β-actin**	Sense: CGAGTACAACCTTCTTGCAG
	Antisense: TGAAGAATGGTCTTGGGTCTTT

### Assessment of microRNA expression

Extraction of microRNAs (miRs) from rat retinas was performed by following the miRNeasy extraction method (Qiagen, Germantown, MD). MiR34a expression was then quantified using miRCURY LNA Universal RT microRNA PCR (Exiqon, Woburn, MA). This system combines a Universal RT reaction, a primer set for miR-103a-3p, an endogenous control, with LNA-enhanced PCR primers designed by the company for the target sequence of miR34a (ACAACCAGCTAAGACACTGCCA; catalog #204486).

### In situ hybridizations (ISH) of miR34a

ISH was performed on frozen retinal section fixed in 4% PFA. MiRs were demasked by incubation with proteinase K for 30 minutes. Slides were incubated overnight at 58°C with a double- (5’ and 3’)-digoxigenin (DIG)-labeled probe for the senescence-associated microRNA 34a (/5DigN/ACAACCAGCTAAGACACTGCCA/3Dig_N/; hsa-miR-34a; Exiqon, Woburn, MA). Slides were then washed in 2x, 1x and .1x concentrations of sodium citrate (SSC) buffers at 58°C, 53°C, and 37°C, respectively, followed by a one hour incubation with anti-DIG (Roche Diagnostics, Indianapolis, IN) and mounting with Fluoromount. Images were captured by light microscopy using Zeiss Axioplan2.

### Lipid Peroxidation assay

Hydroperoxide levels were measured using a quantitative extraction method (Cayman Chemical, Ann Arbor, MI). Lipid hydroperoxides of retinal extracts (300 μg) from control, aging and diabetic retinas were extracted into a degassed chloroform/methanol mixture. Thiocyanate was utilized as the chromogen for detection of hydroperoxide interaction with ferric ions. Absorbance was read at 500nm (spectraMax Gemini EM, Molecular Devices, Sunnydale, CA).

### Statistical analysis

Group differences were evaluated using ANOVA and results were considered significant when p<0.05. For *in vivo* studies, age-matched controls were compared to diabetic rats and aging rats, and diabetic to aging rats (n = 6). Power calculations revealed that when n = 6, the power value was above 0.9 with 50% differences in the treatment effects and a probability of error of 0.05.

### Ethics statement

All animal procedures were performed following the recommendation of the Association for Research in Vision and Ophthalmology (ARVO) Statement for the humane use of animals in vision science and in compliance with the institutional protocols approved by the Animal Care and Use Committee of Georgia Regents University (IACUC#2009–181). All experiments were conducted with the ethical approval of the Institutional Animal Care and Use Committee of Georgia Regents University.

## Results

### Assessment of SA-β-Gal in diabetic and aging retina

**Fig 1:**
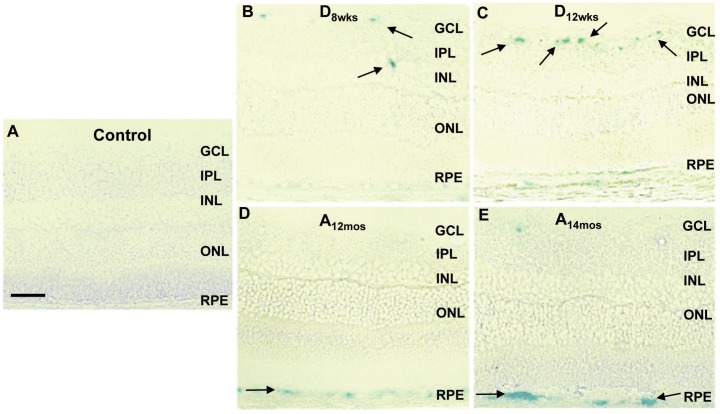
SA-β-gal staining in cross sections of aging and STZ-rat retinas. SA-**β**-gal (blue color) was detected using immunohistochemical analysis of 20 **μ**m frozen retinal sections from 4.5 months old normoglycemic controls (**A**), STZ-rats, at 8 (**B**) and 12 weeks (**C**) of hyperglycemia, and normoglycemic aging rats at 12 (**D**) and 14 (**E**) months of age. Images were captured at 20X magnification. Scale bar equal to *50* **μ** *m*. Black arrows are pointing areas of positive reactivity for SA-**β**-gal (blue color).

We have measured the effects of hyperglycemia in up-regulating SA-**β**-Gal activity at pH 6.0 in retinal tissue. Fig 1A–1E demonstrates the presence of SA-**β**-Gal activity in retinal frozen sections, after 8 (Fig 1B) and 12 (Fig 1C) weeks of hyperglycemia, and in comparison to retinas of aging normoglycemic rats at 12 (A_12_) and at 14 (A_14_) months of age (Fig 1D and 1E, respectively) and to retinas of 4.5 month old normoglycemic control rats (Fig 1A). These images demonstrate a progressive increase in SA-**β**-Gal activity levels in diabetic retinas in comparison to control groups (Fig 1A–1C). In addition, SA-**β**-Gal activity appears to be increased primarily in the inner retina around blood vessels (Fig 1B and 1C, black arrows). Interestingly, retinas of aging rats also showed increased SA-**β**-gal activity, but mainly at the level of the retinal pigmented epithelium (RPE) (Fig 1D and 1E, black arrows).

**Fig 2:**
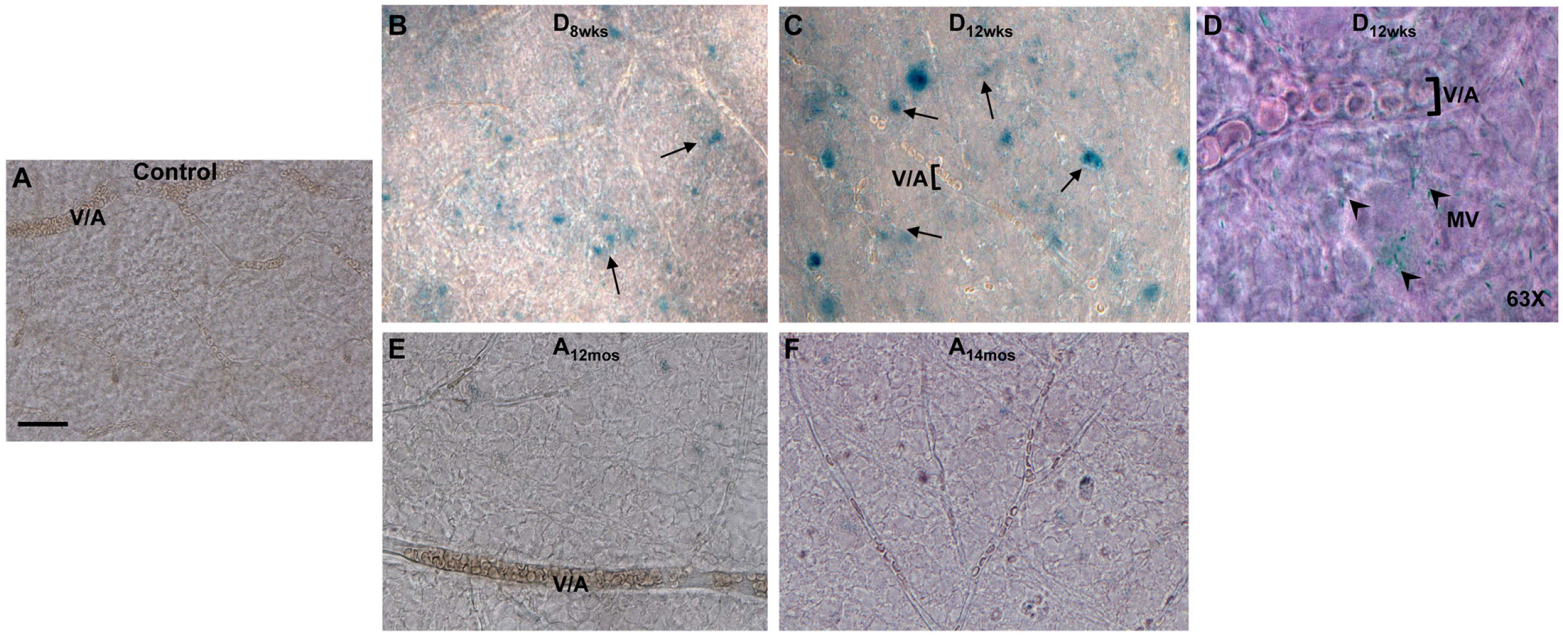
SA-β-gal staining in inner retinal flat mounts. Positivity of SA-**β**-gal at pH6 (black arrows) was assessed in retinal flat mounts from 4.5 month old normoglycemic controls (**A**), STZ-rats at 8 weeks (**B,D**) and 12 weeks (**C**) of hyperglycemia and in non-diabetic aging rats at 12 (**E**) and 14 (**F**) months of age. In panel **D**, SA-**β**-gal reactivity is showed at 63X magnification to demonstrate positive reactivity in microvascular structures in the diabetic retina (8 weeks of hyperglycemia, black arrowheads). MV = microvascular vessels, V/A = venules/arterioles. Scale bar equal to *20* **μ** *m*.

To confirm the presence of SA-**β**-Gal activity in the vasculature of the diabetic retina and in the RPE layer in the aging rats, we have assessed this senescence marker in flat-mounted retinas and isolated retinal pigmented epithelium layers. Flat mount images in Fig 2 show increased SA-**β**-Gal activity in the vasculature of the diabetic retina at 8 and 12 weeks of hyperglycemia (black arrows in Fig 2B and 2C, respectively) in comparison to normoglycemic 4.5 months old controls (Fig 2A) and normoglycemic aging rat retinas at 12 and 14 months of age (Fig 2E and 2F, respectively).

**Fig 3:**
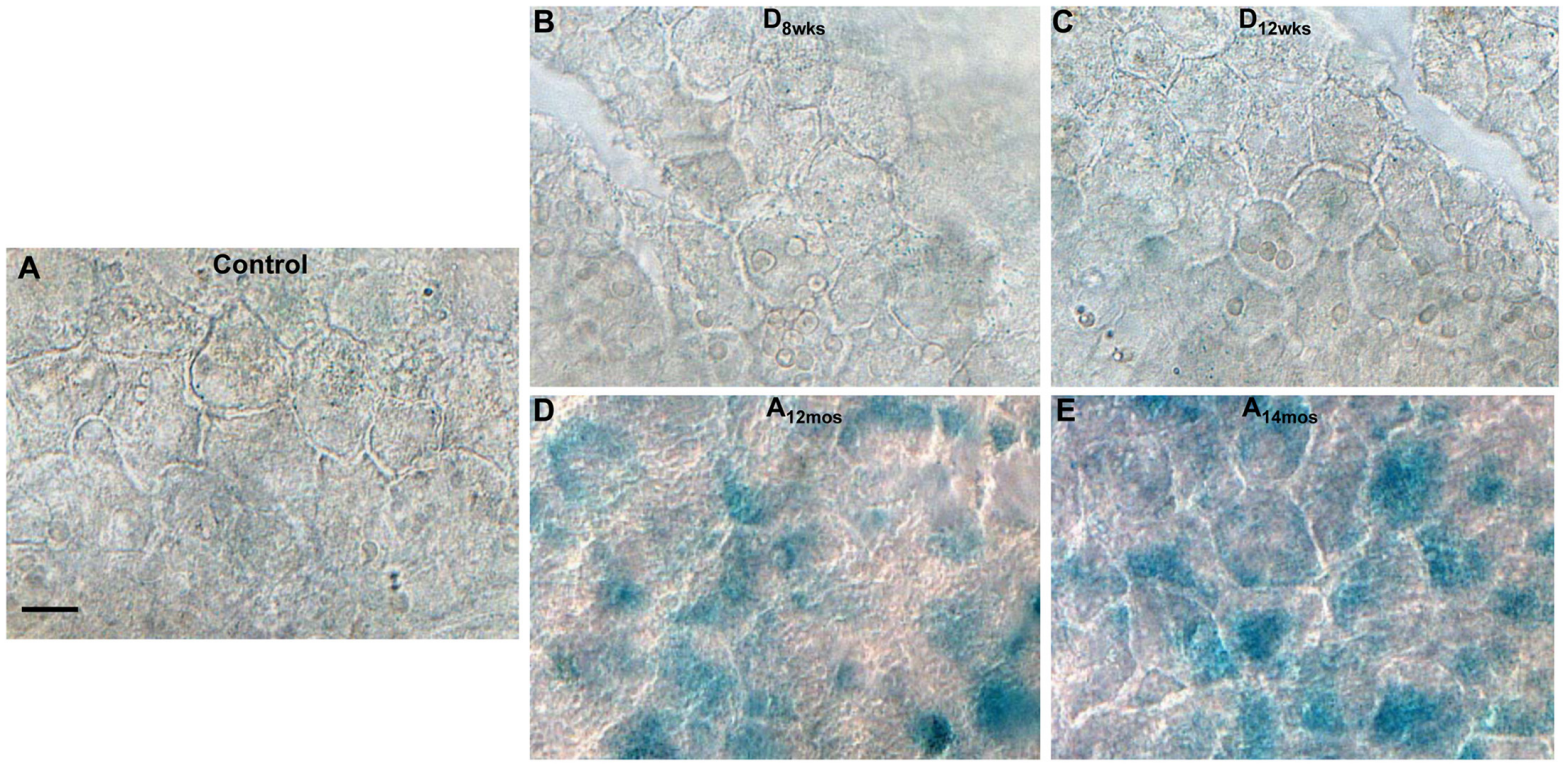
SA-β-gal in RPE flat mounts. Bright-field images show SA-**β**-gal positive areas found in RPE flat mounts from age-matched (4.5 months old) normoglycemic control rats (**A**),STZ-rats, at 8 (**B**) and 12 weeks (**C**) of hyperglycemia, or in normoglycemic aging rats at12 (**D**) and 14 (**E**) months of age. Scale bar equal to *20* **μ** *m*.

Interestingly, higher magnifications indicate that SA-**β**-Gal activity is particularly evident microvasculature structures, which include capillaries and postcapillary venules (black arrowheads, Fig 2D) in comparison to larger arterioles and venules. Flat-mounted RPE layers showed higher SA-**β**-Gal activity in aging normoglycemic (Fig 3D and 3E) rat retinas than that measured in diabetic (Fig 3B and 3C) or non-diabetic (4.5 months old) control rat retinas (Fig 3A).

### SIRT-1 expression and activity in the diabetic and aging retina

**Fig 4:**
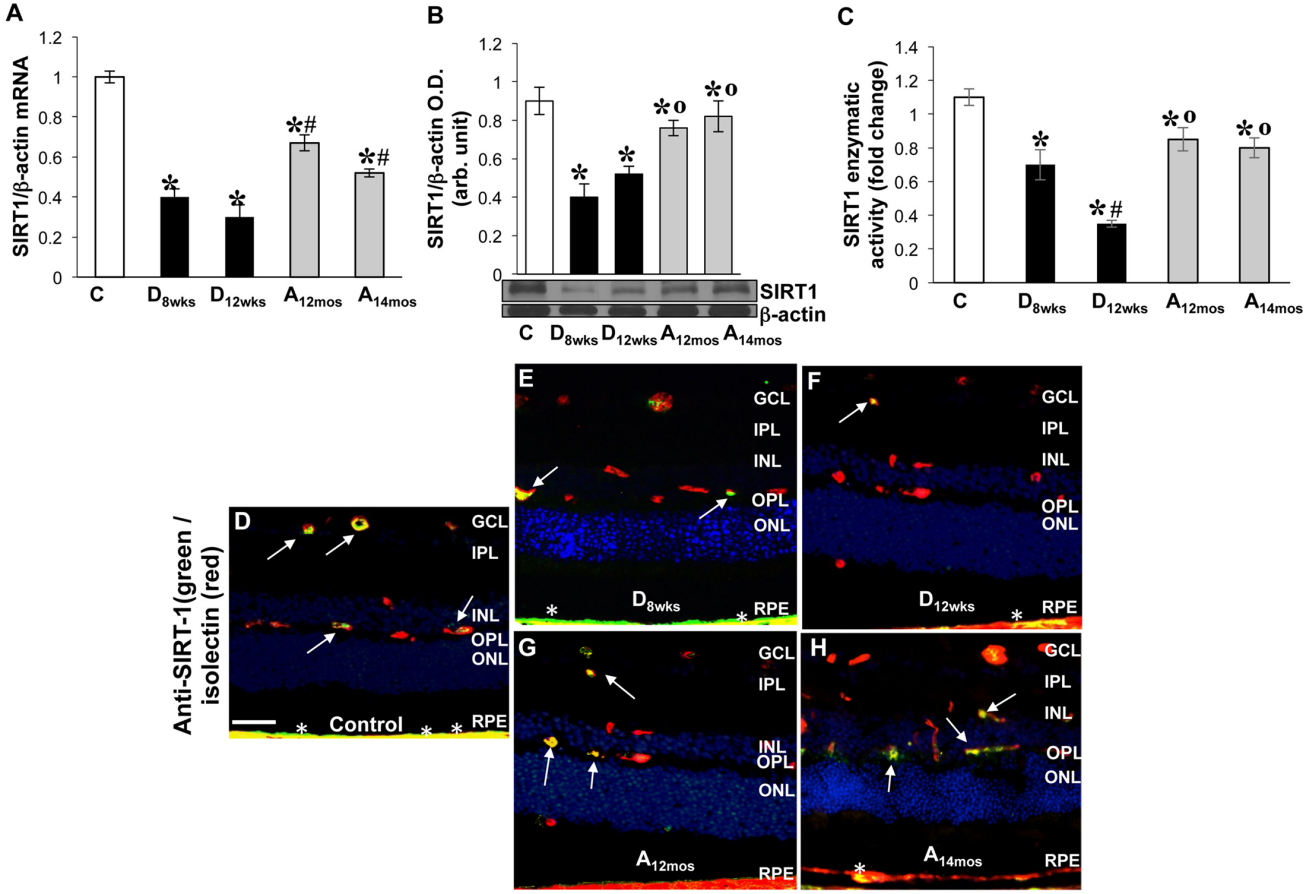
SIRT-1 expression and activity in rat retinas. **A)** Expression of SIRT-1 at mRNA level was measured using qPCR in retinal extracts from control, diabetic, and aging rat retinas. MRNA levels were calculated as a ratio to **β**-actin expression and normalized to baseline controls. x± S.D, *p<0.02 *vs* C rat retina; #p<0.02 *vs* D_8wks_ diabetic; **°**p<0.05 *vs* D_12wks_ diabetic n = 6. **B)** Bar histogram showing SIRT-1 protein levels normalized to **β**-actin in retinal extracts. x ± S.D.,*p<0.04 vs control rat retina; #p<0.02, n = 6. **C)** Changes in enzymatic activity of SIRT-1 *in vivo* are displayed in bar histogram. x ± S.D, *p<0.05 *vs* control rat retina; #p<0.02 *vs* D_8wks_, n = 6. Control retinas = white bar; aging retinas = gray bar; diabetic retinas = black bar. **D-H)** Retinal frozen sections were probed with anti-SIRT1 (*green*) and anti-isolectin (*red*) to detect SIRT-1-specific immunoreactivity in control (**D**), diabetic (**E-F**), and aging (**G-H**) rats. White arrows are indicating areas of merging double labeling (yellow) in inner blood vessels. White asterisks indicate areas SIRT1 immunoreactivity at the RPE/choroid level. Hoescht staining was used to detect nuclei (*blue*). Scale bar equal to *50* **μ** *m*.

Vascular senescence has been associated with decreased expression of SIRT-1 in vascular cells [18, 37]. We have conducted studies to assess SIRT-1 expression and activity in the diabetic retinas. As shown in Fig 4, SIRT-1 expression, at both mRNA [Fig 4A, *p<0.02 *vs* control (C), ^#^p<0.02 *vs* D_8wks_, n = 6)] and protein level (Fig 4B, *p<0.04 *vs* C) along with its enzymatic activity (Fig 4C, *p<0.05 *vs* C, °p<0.05 vs D_8wks_, n = 6) were significantly down-regulated in whole retinal tissue extracts of diabetic rats compared to both control and normoglycemic, aging retinas.

Morphological analysis confirmed the effects of hyperglycemia on SIRT-1 expression in the diabetic retinal microvasculature, as shown by double labeling with isolectin B4, demonstrating a down-regulation of SIRT-1-specific immunoreactivity in retinal blood vessels of diabetic rats (Fig 4E and 4F, white arrows) as compared to control (Fig 4D, white arrows) and aging (Fig 4G and 4H) rat retinas. Notably, SIRT1 immunoreactivity diminished at the RPE/choroid level in aging retinas (asterisk, 4G,H).

### Up-regulation of p16^INK4A^expression in the diabetic and aging retina

Senescence promotes the withdrawal of vascular cells from the cell cycle due to up-regulation of cyclin-dependent kinase inhibitors such as p16^INK4A^ [17, 38]. Furthermore, studies have shown that p16^INK4a^ can also be activated in response to oxidative stress, through the action of the p38-MAPK protein [39].

**Fig 5:**
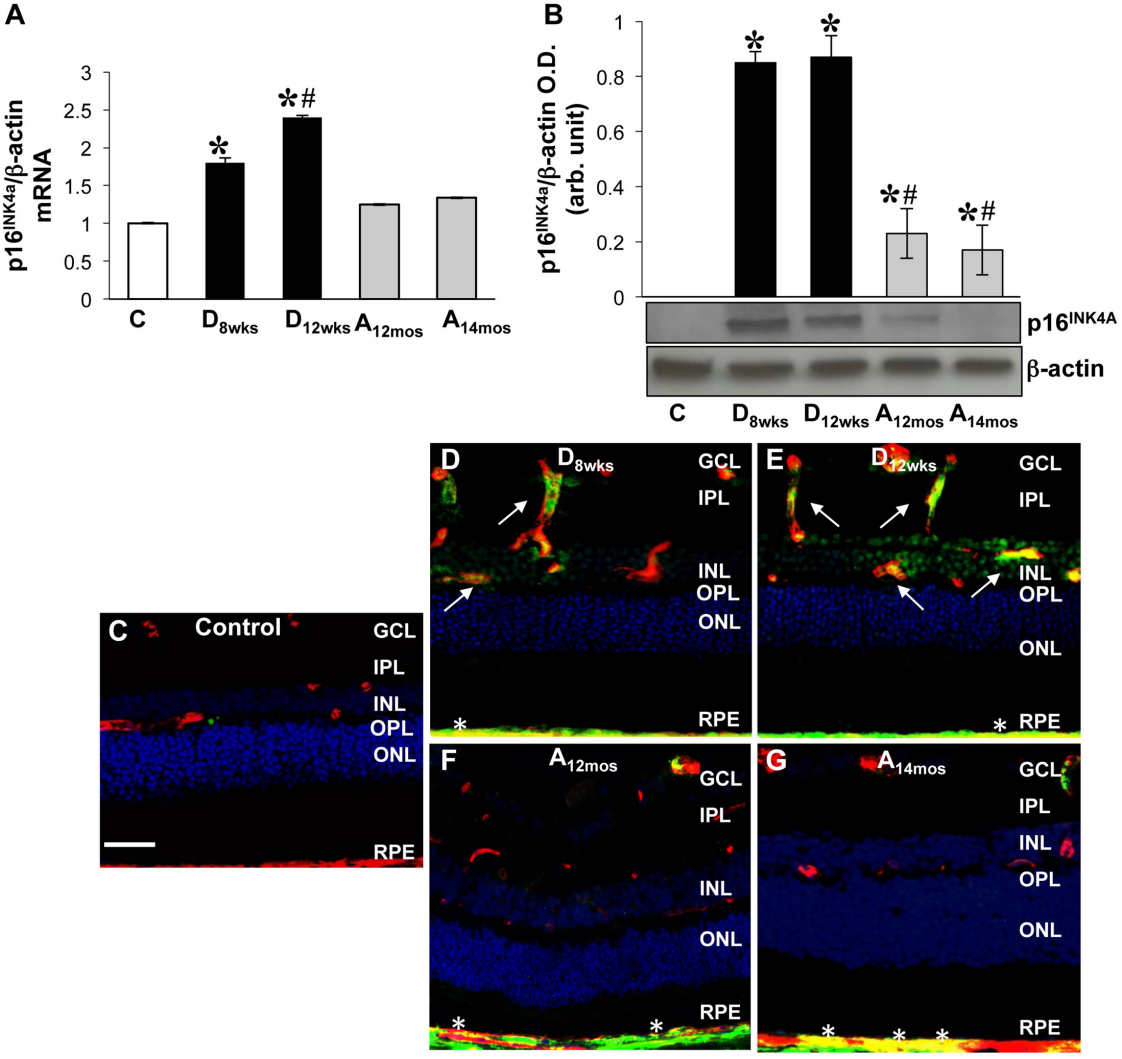
Measurements of p16^INK4a^ levels in rat retinas. **A)** Expression of p16^INK4a^ at mRNA level was measured using qPCR in retinal extracts from control, STZ, and aging rat retinas (as indicated above). Levels of p16^INK4A^ specific mRNA are expressed as a ratio to **β**-actin and normalized to baseline controls. x ± S.D, *p<0.009 vs control 4.5 month rat retina; #p<0.01 *vs* D_8wks_, n = 6. **B)** Western blotting analysis measuring p16^INK4a^ protein levels; bar histogram depicts p16^INK4a^ protein levels normalized to **β**-actin in retinal extracts. x ± S.D,*p<0.01 *vs* C; ^#^p<0.04 *vs* D_8wks_ diabetic, n = 6. Control retinas = white bar; aging retinas = gray bar; diabetic retinas = black bar. **C-G)** Frozen retinal sections were probed with anti-p16^INK4a^ (*green*) antibodies and isolectin B4 (*red*) to detect anti-p16^INK4a^ -specific immunoreactivity in retinal vessels of control (**C**), diabetic (**D-E**), and aging (**F-G**) rats. Areas of merging labeling (yellow) are indicated by the white arrows. White asterisks show p16^INK4a^ positivity at the RPE/choroid level. Hoescht staining was used to detect cellular nuclei (*blue*). Scale bar equal to 50 **μ**m.

We have measured the expression levels of p16^INK4A^ in diabetic retinas in comparison to those measured in the aging and adult normoglycemic rat retinas. As shown in Fig 5, the expression of p16^INK4A^, measured by qPCR and Western blotting analyses, was progressively and significantly up-regulated in the diabetic retina at both mRNA and protein levels in comparison to aging and control rat retinas [Fig 5A (*p<0.009 *vs* control, ^#^p<0.01 *vs* D_8wks_, n = 6) and Fig 5B (*p<0.01 vs control vs D_8wks_), ^#^p<0.04, n = 6) respectively].

Immunohistochemical analysis further revealed that p16^INK4A^ was specifically up-regulated in the retinal vasculature of both 8 and 12 weeks of hyperglycemia (Fig 5D and 5E), in comparison to adult normoglycemic controls (Fig 5C). Non-diabetic aging rat retinas showed increased immunoreactivity to p16^INK4A^ at the RPE/choroid interface, thus, mirroring the SA-**β**-Gal results observed in Fig 1 (Fig 5F and 5G, asterisks). This supports findings that mature RPE cells have the capacity to replicate, and therefore, can develop a senescent phenotype [40].

### Expression of miR34a in the diabetic and aging retina

**Fig 6:**
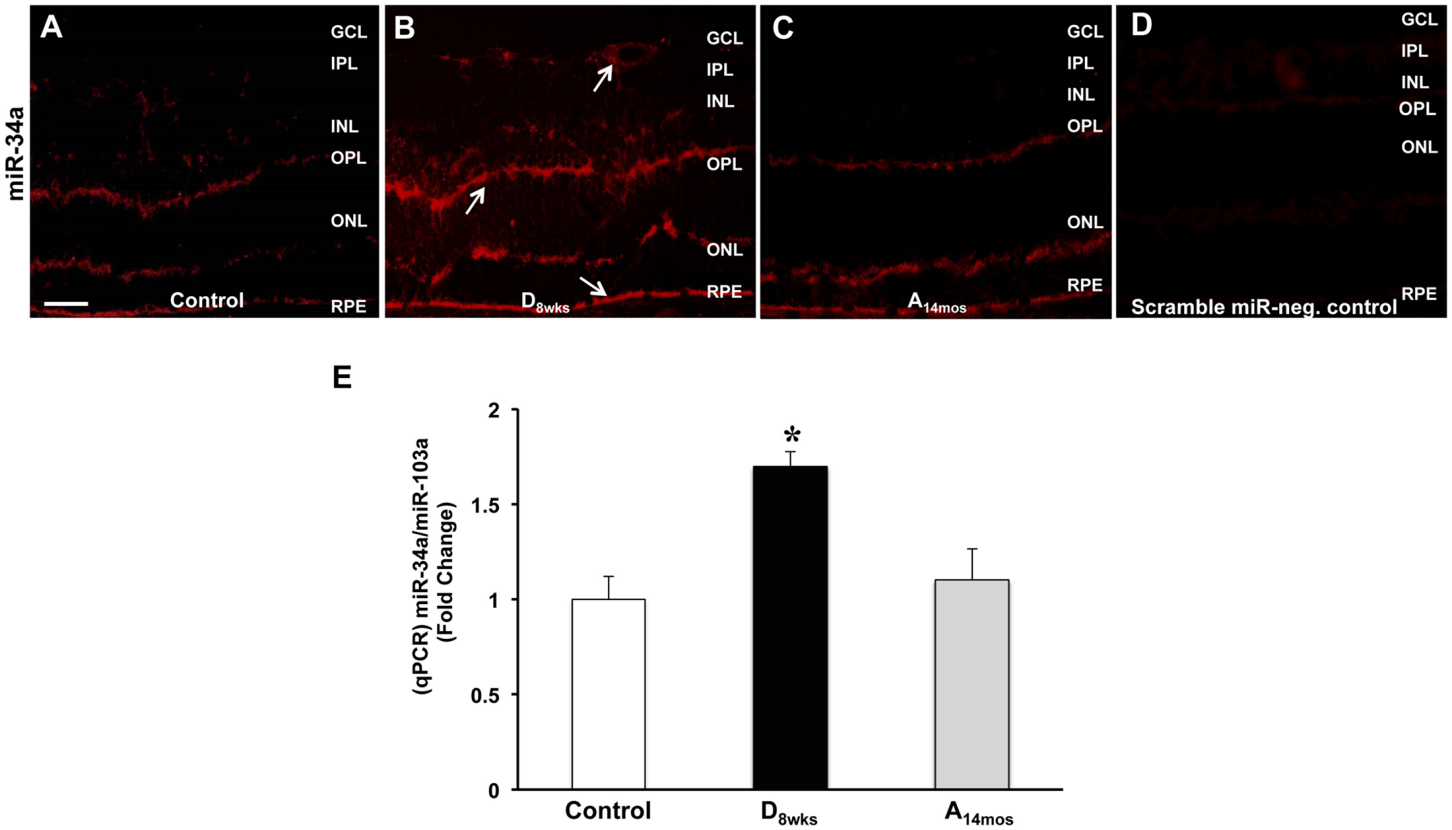
Assessment of miR34a expression. MiR-34a detection was evidenced by *in situ* hybridization in retinal sections of control (4.5 months old), 8 wks diabetic, and 14 months old aging rats. Representative images of control (**A**), diabetic (**B**), and aging (**C**) rat retinas probed for MiR-34a-DIG are depicted in each panel. **D)** Control retina probed with scrambled miRNA. Scale bar *50* **μ** *m*. In **E**) bar histogram representing relative fluorescence units of miR34a amplicons measured by qPCR.x ± S.D, *p<0.01 *vs* C 4.5 month rat retina, n = 6.

Evidence is provided demonstrating that vascular senescence involves epigenetic changes including up-regulation of miR34a [41]. To further analyze the effects of hyperglycemia on accelerated vascular senescence, we have assessed miR34a expression and localization in retinal sections via a highly specific *in situ* hybridization (ISH) technique for miRNA detection in tissue samples. Images in Fig 6 indicate that miR34a reactivity is up-regulated in the diabetic retina (Fig 6B) as compared to normoglycemic aging (Fig 6C) and control adult retina (Fig 6A).

ISH revealed that miR34a expression is localized around retinal blood vessels (white arrow), but also in other cells of the inner retina as well as in the RPE of the diabetic retinas (Fig 6B).

Quantification of miR34a expression by qPCR (Fig 6D) further confirmed the results of the ISH by showing a significant increase in total retinal expression of miR34a in the diabetic retinas as compared to control, normoglycemic, 4.5 month old rat retinas (*p<0.01 *vs* control). Retinal expression of miR34a, measured by qPCR, was also found to be slightly increased in the aging retinas, however this was not statistically different from the normoglycemic adult control (Fig 6D).

### Effects of hyperglycemia and aging on levels of oxidative and nitrative stress markers

One of the main inducer of accelerated vascular cell senescence is the up-regulation of reactive oxygen and nitrogen species (ROS and RNS, respectively) and consequent oxidative and nitrative/stress [42–45]. Therefore, we have conducted a comparative analysis of the levels of oxidative and nitrative stress markers in the retinas of diabetic rats in comparison to normoglycemic aging or adult rats.

**Fig 7:**
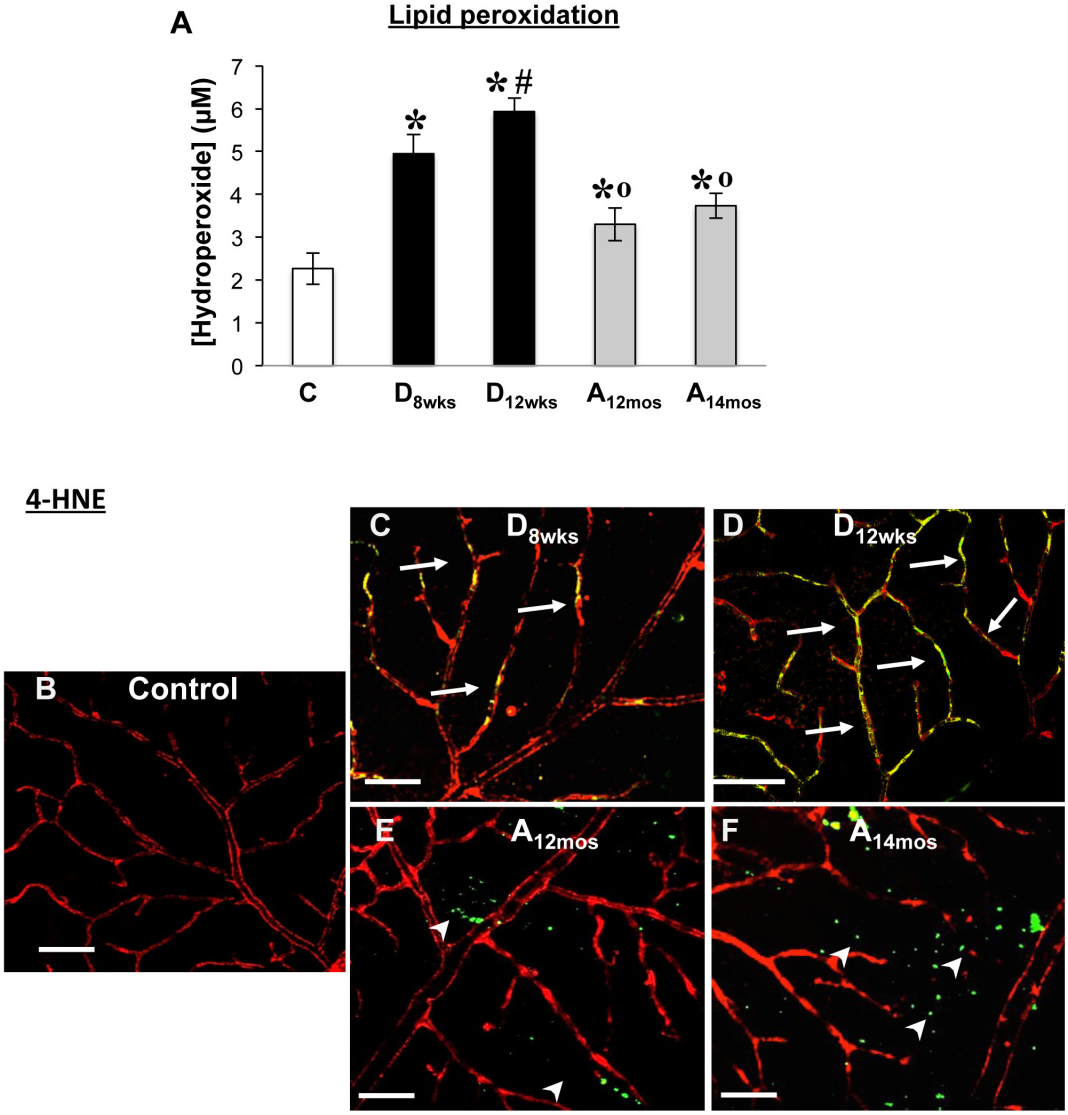
Measurements of lipid oxidative modifications. Bar histogram is representative of measurements of chloroform/methanol extracted hydroperoxides (**A**) in retinal rat tissues in the different treatment groups. x ± S.D, *p<0.0001 *vs* C, ^#^p<0.0001 *vs* D_8wks_, °p<0.0001 vs D_8wks_, n = 6. Immunohistochemical analysis using anti-4-HNE specific antibodies in retinal flat mounts is shown in panels B and C. Areas of merging labeling (yellow) are indicated by the white arrows. White arrowheads indicate extravascular areas immunoreactive to 4-HNE. Scale bar equal to *20* **μ** *m*.

**Fig 8:**
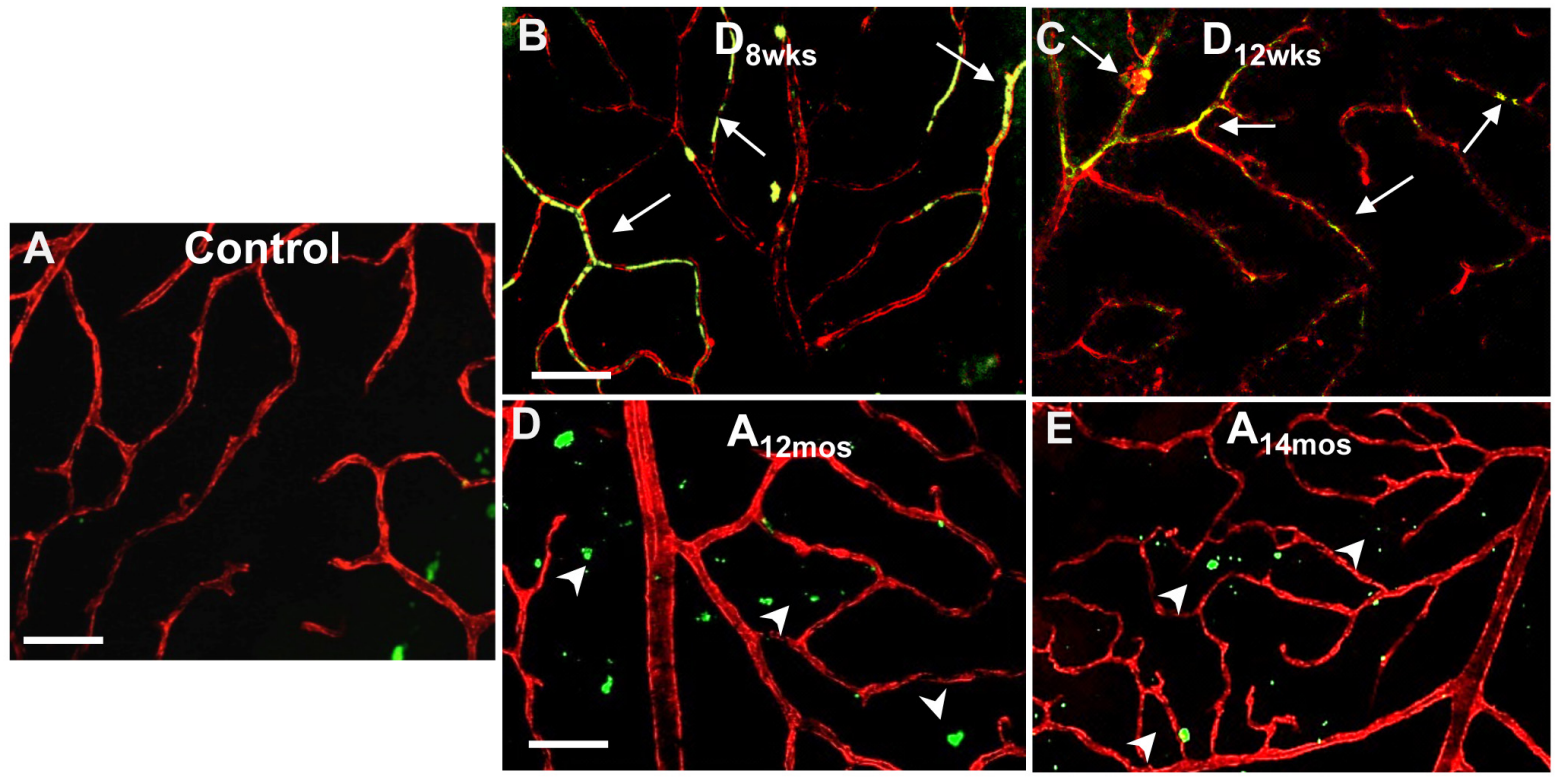
Measurements of nitrative modifications. Representative images of immunohistochemical analysis using anti-nitrotyrosine in retinal flat mounts to demonstrate changes in retinal NY formation in the different treatment groups. Double labeling with isolectin B4 was used to specifically assess NY immunoreactivity in the retinal microvasculature. Areas of merging labeling (yellow) are indicated by the white arrows. Non-vascular areas immunoreactive to NY are indicated by white arrowheads. Scale bar equal to *20* **μ** *m*.

Levels of lipid peroxidation, determined by assessing hydroperoxide concentrations in total retinal lysates, were increased in STZ-rats at 8 and 12 weeks of hyperglycemia with levels detected at 12 weeks of hyperglycemia being significantly higher than the diabetic at 8 weeks of hyperglycemia (Fig 7A, *p<0.0001 *vs* C, ^#^p<0.0001 *vs* D_8wks_, n = 6). In aging retinas of 12 and 14 months old rats, we have detected a significant increase in hydroperoxides levels compared to control rats (Fig 8A, *p<0.05 *vs* C, n = 6), however this value was significantly lower than that found in the diabetic retinas (Fig 7A, p<0.05 *vs* D_8wks_, n = 6).

Measurements of 4-hydroxynonenal (4-HNE) in retinal flat mounts, to assess aldehydic products of lipid peroxidation, showed increased adduct formation specifically in the retinal vasculature of diabetic rats at 8 and 12 weeks of diabetes (Fig 7C and 7D respectively, white arrows), whereas in retinal flat mounts of 12 and 14 month-old (aging) rats we found immunoreactivity in extra-vascular areas of the retina (Fig 7E and 7F, white arrowheads).

We have also determined the occurrence of protein tyrosine nitration in the different experimental groups. We have assessed nitrotyrosine formation in control, aging and diabetic rat retinal flat mounts (Fig 8). Virtually no detection of nitrotyrosine-specific immunoreactivity was observed in control retinas (Fig 8A) while this was increased in vascular structures of diabetic rats after 8 and 12 weeks of hyperglycemia (Fig 8B and 8C, respectively). Nitrotyrosine formation in retinal flat mounts of aging rats was also increased compared to control retina, but in a lesser extent than in diabetic retinas and was mainly localized in extra-vascular areas (Fig 8D and 8E, white arrowheads).

The sum of these results show that oxidative and nitrative retinal injury (lipid peroxidation and nitrotyrosine adducts formation) was more pronounced in the diabetic retinal vasculatures than in control and aging retina where was present in extravascular structures.

### Effects of FeTPPS on vascular senescence in diabetic retinas

The previous data show that increased formation of nitrotyrosine adducts occurs in the diabetic retina. As nitrotyrosine formation is directly linked to production of the free radical peroxynitrite (ONOO-), we have tested the effects of the ONOO- decomposition catalyst 5,10,15,20-tetrakis(4-sulfonatophenyl) porphyrinato iron III chloride (FeTPPS) to determine whether scavenging of ONOO-, by limiting oxidative and nitrative stress, could also prevent hyperglycemia-induced vascular senescence.

**Fig 9:**
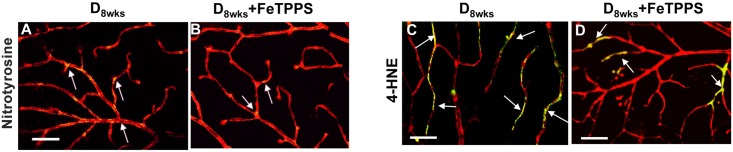
FeTPPS effects on lipid peroxidation and nitrotyrosine formation. Representative images of anti-nitrotyrosine (**A,B**) and anti-4-HNE (**C,D**) immunoreactivity (*green*) in retinal flat mount preparations of STZ-diabetic rats in comparison to retinas of diabetic rats that were treated with FeTPPS. Double labeling for isolectin B4 (*red*) was performed to visualize merging areas (yellow) corresponding to vascular structures. Scale bar equal to *20* **μ** *m*.

**Fig 10:**
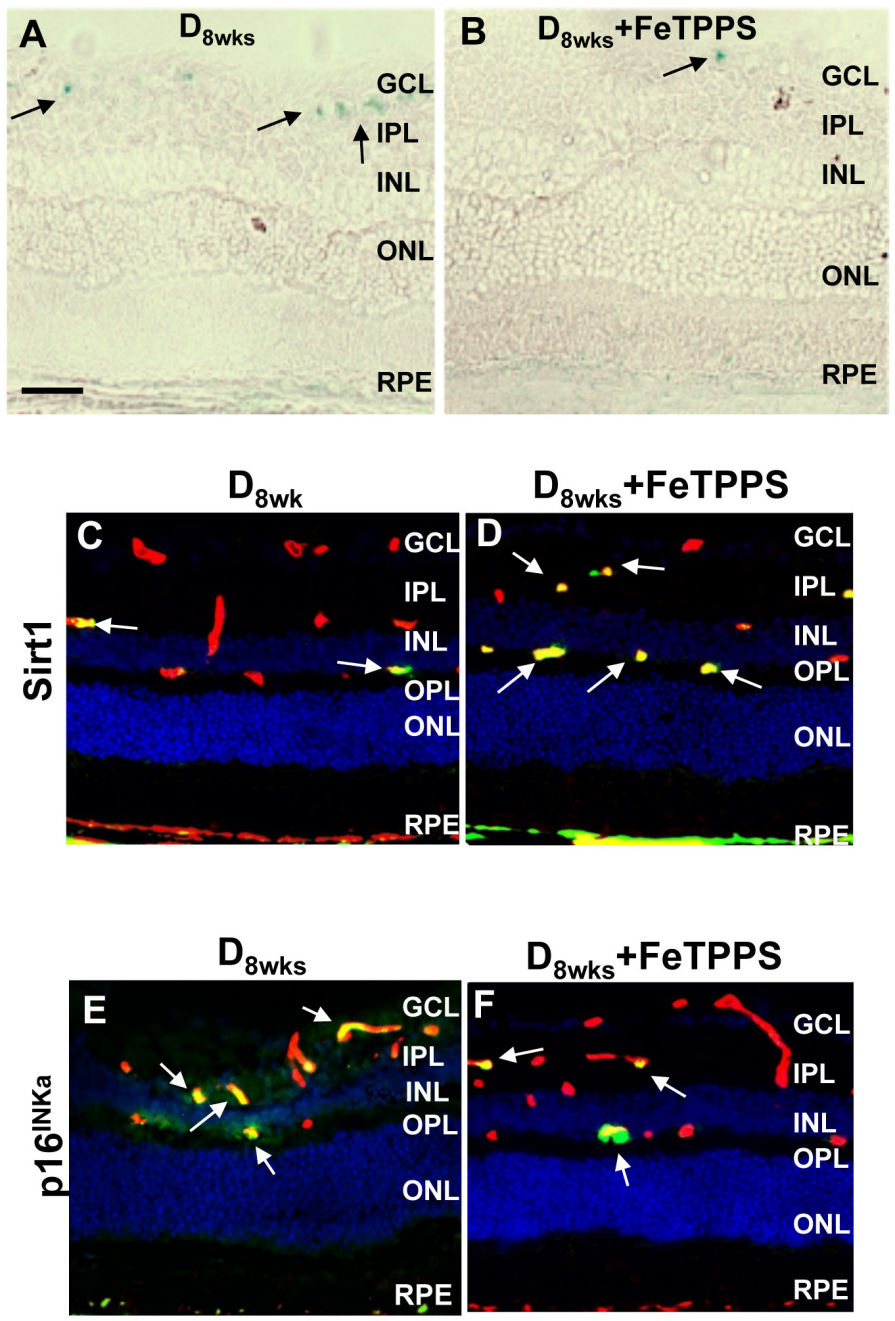
Senescence-associated retinal changes with FeTPPS treatment. **A,B**) Retinal frozen sections from diabetic and diabetic + FeTPPS groups were probed at pH6 for detection of SA-**β**-gal positivity (*blue*, *black arrows*). Representative images from frozen retinal sections probed with anti-SIRT1 (**C, D**) and anti-p16^INK4a^ (**E, F**) (*green*) to detect immunoreactivities in diabetic and retinas of FeTPPS- treated diabetic rats. Sections were co-labeled with anti-isolectin B4 (vascular structures, *red*). Hoescht staining was used to detect nuclei (*blue*). Scale bar equal to 50**μ**m.

Treatments with the ONOO- decomposition catalyst FeTPPS significantly diminished the levels of nitrotyrosine formation (Fig 9A and 9B, white arrows) and 4-HNE (Fig 9C and 9D, white arrows) in the diabetic retina. FeTPPS treatment also decreased SA-**β**-Gal reactivity detected in retinal frozen sections (Fig 10A and 10B) of diabetic rats. Examination of the senescence markers, SIRT1 and p16^INK4a^, revealed that FeTPPS-treated diabetic rats displayed increased/restored SIRT1-specific immunoreactivity (Fig 10C and 10D, white arrows) and also decreased p16^INK4a^–specific immunoreactivity in inner retina blood vessels (Fig 10E and 10F, white arrows).

## Discussion

In this study we have investigated the effects of hyperglycemia in promoting retinal vascular cell premature aging, a molecular event shown to lead to vascular dysfunction and induction of inflammatory processes, all factors involved in the pathogenesis of DR [10, 20].

Our data showed that in a rat model of type 1 diabetes (STZ-rat) there was acquisition of senescent-like phenotype of the retinal microvasculature demonstrated by an up-regulation of senescence-associated markers.

Cellular senescence can be divided into two categories: replicative senescence, which is dependent on the number of spontaneously completed cell divisions, and stress-induced premature senescence (SIPS) [23, 46]. The latter is suggested to be independent of telomere status [47] and can be accelerated by a number of stimuli, including oxidative stress [48–51].

SA-**β**-gal activity at pH 6 occurs only in senescent cells as a result of the accumulation of endogenous lysosomal beta-galactosidase [52]. In retinal flat mounts, higher SA-**β**-gal reactivity was evidenced in the microvasculature of STZ-rats (Fig 2), whereas in the aging rats was mainly accumulated in the RPE (Fig 3).

Up-regulation of post-mitotic factors such as the cyclin-dependent kinase inhibitor p16^INK4A^ [53], can be found in blood vessels in response to redox-dependent induction of p53 [54] and downstream activation of p38MAPK [55]. Increased expression of p16^INK4A^ at mRNA and protein levels was detected in the diabetic retinas at significantly higher levels than retinas of aging and control age-matched rats. Immunohistochemical analysis, conducted in retinal sections and flat mounts, [56] confirmed that p16^INK4A^ expression was primarily localized in retinal blood vessels of diabetic rats (Fig 5).

Blunted expression and activity of the histone deacetylase SIRT-1 was also evidenced in the retinal microvasculature of diabetic rats (Fig 4). Loss of SIRT-1 deacetylase activity results in persistent acetylation/activation of the transcription factor NF-kB and consequent expression of pro-inflammatory genes [56–58]. Therefore, loss of SIRT-1 may explain hyperglycemia-induced chronic inflammatory responses in the diabetic retina [59].

Suppression of SIRT-1 expression has been shown to be regulated by epigenetic mechanisms such as up-regulation of miR34a [41, 60]. In our experimental conditions, retinal levels of miR-34a were significantly elevated in the diabetic rats as compared to control adult and aging rats. Interestingly, *in situ* hybridization showed that miR34a is also produced in non-vascular retinal cells (Fig 6A–6C). MiRs can be released in exosomes, thus, affecting gene expression in an autocrine and paracrine manner [61, 62]. In addition, mirR34a is p53-dependent [63] and its expression can also be triggered by oxidative and nitrative stress. Our data, therefore, suggest the interesting possibility that in the hyperglycemic milieu different retinal cells can contribute to the induction of retinal microvascular cell senescence through stress-related expression and release of miR34a.

Our data showed that hyperglycemia-induced up-regulation of senescence-like markers appears to be a consequence of oxidative/nitrative stress and treatments of the diabetic rats with the ONOO- decomposition catalyst, FeTPPS, significantly prevented the appearance of senescence markers in the diabetic retinal microvasculature (Figs 9 and 10).

In addition, our study revealed that aging retinas express senescence markers primarily in the RPE/choroid layer, an observation tangential to our focus on retinal vascular beds affected in DR. Whether RPE cells are post-mitotic or not remains controversial. However, recent studies demonstrate that regional proliferation differences exist in adult RPE cells [40, 64, 65].

In summary, the results of the present studies demonstrate that hyperglycemia-induced oxidative/nitrative stress accelerates retinal vascular cell senescence and consequent pro-inflammatory processes, thus, providing new relevant information on the pathogenesis of diabetic retinal microangiopathy and DR progression.
